# Protocol for performing and analyzing a live-cell imaging-based microglia phagocytosis assay using AI on the Olympus ScanR system

**DOI:** 10.1016/j.xpro.2025.103846

**Published:** 2025-07-15

**Authors:** Alexander Zähringer, Janaki Manoja Vinnakota, Tobias Wertheimer, Marie Follo, Robert Zeiser

**Affiliations:** 1 Department of Medicine I, Medical Center-University of Freiburg, Faculty of Medicine, Albert-Ludwigs-University Freiburg, Hugstetter Straße 55, Freiburg 79106, Germany

**Keywords:** Cancer, Health Sciences, Microscopy, Neuroscience

## Abstract

Various neurological disorders show dysregulation of microglial function, including phagocytosis, necessitating accurate and efficient analysis. Here, we present a protocol using our custom-trained neural network (AIstain) in the Olympus ScanR environment for easy-to-use workflows of microglial phagocytosis assays. We describe steps for preparing microglia and setting up the microscope. We then explain the workflow of live-cell imaging and usage of AI in the data analysis. This protocol facilitates the preparation of phagocytosis assays and improves accuracy in data analysis.

For complete details on the use and execution of this protocol, please refer to Zähringer et al.^1^

## Highlights

• Label-free live-cell imaging approach to investigate microglial phagocytosis
• Steps for preparing the cell culture for high-throughput analysis
• Detailed instructions for microscope setup, software, and analysis
• Pre-configured analysis assay utilizing AIstain (U-Net-based neural network) provided

## Abstract (editor highlights)

Publisher’s note: Undertaking any experimental protocol requires adherence to local institutional guidelines for laboratory safety and ethics.

## Graphical abstract

**Figure:**
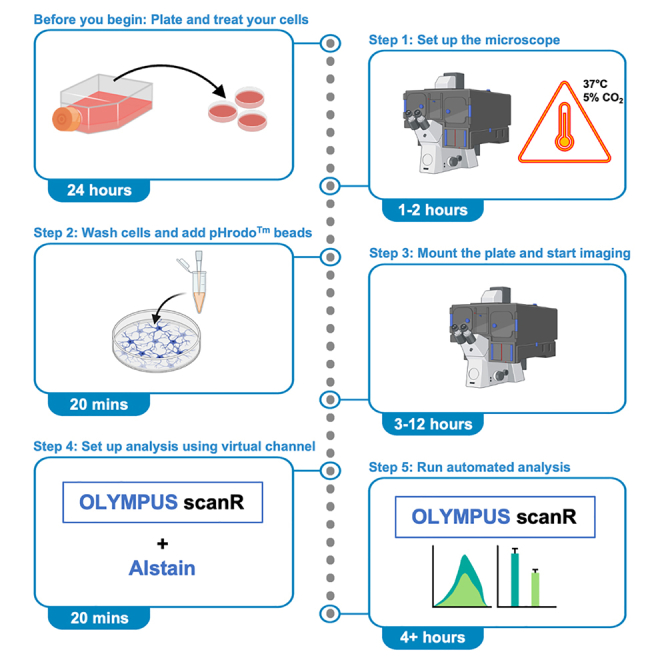

## Before you begin

**Timing: 0.5–2 h**

This protocol was used to analyze microglial phagocytosis in live cell imaging over 3.5 h.

***Note:*** The protocol below describes the specific steps for using our provided neural network (AIstain) in live cell imaging and phagocytosis analysis of primary microglia. However, the neural network can also be used for the detection of fixed cells, see note below at the respective analysis step.

***Note:*** AIstain is a custom-designed and trained U-Net based neural network. AIstain was trained within the Olympus ScanR analysis software v.3.4.1.

***Note:*** AIstain is only used for cell identification and segmentation. Apart from these steps, AIstain does not interfere with parameter computation.

***Note:*** The protocol below describes the specific steps for using primary murine microglia. However, other phagocytosing cells can be used as well. Details on necessary adaptions for other cell types can be found in the section “adaptions for other cell types” below.

**CRITICAL:** The neural network-based analyses and assays are computationally intensive and require at least the following computational resources, which we used for performing the analyses. Less powerful systems might work as well but may result in increased analysis time.

  Processor: Intel Xeon W-2255 CPU 3.70 GHz.

  RAM: 64.0 GB.

  System: 64 Bit.

  Graphic card: NVIDIA Quadro RTX 4000.

  Dataset was saved on institutional network server.

**CRITICAL:** Make sure that you prepare and handle all reagents and substances sterile.

1. Prepare pHrodo BioParticles Conjugates for phagocytosis by dissolving the lyophilized product according to manufacturer’s instructions.
2. Once dissolved, pHrodo BioParticles Conjugates for phagocytosis should be stored at 4°C for a maximum of 3 months.
3. Prepare FluoroBrite Medium by adding 10% FCS and other supplements if necessary.

**CRITICAL:** Make sure that your medium does not contain any fluorescent substances as these will interfere with your experiment.

4. One or two days before the experiment, plate 10.000 cells/well of your microglia or cells of interest in a 96-well plate with glass bottom and start the pretreatment of your cells.
5. On the day of the live cell imaging, pre-warm the reagents to 37°C.

***Note:*** Your plate and/or your cells may require pre-coating of the plate to achieve adherence of the cells. Check this before you plate the cells or run a first trial to determine optimal conditions.

### Institutional permissions

Animal protocols (Protocol numbers: X-20/07A, X-20/06K, X-15/10A) were approved by the Regierungspräsidium Freiburg, (regional council), Germany (Federal Ministry for Nature, Environment and Consumers Protection).

Primary microglia were derived from female C57BL/6 mice. The mice were purchased either from Janvier Labs (France) or from the local stock of the animal facility at the University of Freiburg and maintained at the animal facility at the University of Freiburg. Mice were used between 6 and 14 weeks of age.

**CRITICAL:** Any experiments on live vertebrates or higher invertebrates must be performed in accordance with relevant institutional and national guidelines and regulations. You must approve the use of animals and cell lines by the relevant institutions.

**CRITICAL:** Make sure that your lab is allowed to handle specific cell lines and that you wear appropriate protective clothing.

## Key resources table

**Table:** 

REAGENT or RESOURCE	SOURCE	IDENTIFIER
**Chemicals, peptides, and recombinant proteins**		

FluoroBrite DMEM	Gibco Thermo Fisher Scientific	Cat#A1896701
Dulbecco’s phosphate-buffered saline (PBS)	Sigma-Aldrich	Cat#D8537-500ML
Fetal bovine serum South America (FCS)	Anprotec	Cat#AC-SM-0027

**Critical commercial assays**		

pHrodo BioParticles conjugates for phagocytosis and phagocytosis kit, for flow cytometry – deep red	Invitrogen Thermo Fisher Scientific	Cat#P35360

**Deposited data**		

AIstain training data set	This paper	Zähringer, Alexander (2024), “Enhancing microglial phagocytosis analysis through deep learning: A U-Net based approach”, Mendeley Data, V1, https://doi.org/10.17632/czh96my8cf.1

**Experimental models: Cell lines**		

Primary murine microglia obtained from C57BL/6 mice, female, 6–10 weeks old	Generated by the authors of this paper	N/A

**Experimental models: Organisms/strains**		

C57BL/6 mice female, wild-type, 6–10 weeks old	CEMT University Medical Center Freiburg	N/A

**Software and algorithms**		

Olympus ScanR Acquisition 3.4.1 or 3.5 (or higher)	Olympus Life Science Solutions	https://www.olympus-lifescience.com/de/support/downloads/%20/
Olympus ScanR Analysis 3.4.1 or 3.5 (or higher)	Olympus Life Science Solutions	https://www.olympus-lifescience.com/de/support/downloads/%20/
Trained Neural Network AIstain	This paper	Zähringer, Alexander (2024), “Enhancing microglial phagocytosis analysis through deep learning: A U-Net based approach”, Mendeley Data, V1, https://doi.org/10.17632/czh96my8cf.1
AIstain Sample Assay	This paper	Zähringer, Alexander (2024), “Enhancing microglial phagocytosis analysis through deep learning: A U-Net based approach”, Mendeley Data, V1, https://doi.org/10.17632/czh96my8cf.1

**Other**		

Olympus ScanR IX-83 high-throughput microscope	Olympus Life Science Solutions	https://www.olympus-lifescience.com/en/microscopes/inverted/scanr/
Bath sonicator	N/A	N/A
Cellvis 96-well glass bottom plate with high performance #1.5 cover glass	Cellvis	Cat# P96-1.5H-N

## Materials and equipment

**CRITICAL:** All materials require sterile handling.

**CRITICAL:** Check for current manufacturer’s instructions for the correct dilution of pHrodo BioParticles Conjugates.

**Table:** pHrodo BioParticles Deep Red Conjugates

Reagent	Final concentration	Amount
PBS	N/A	2 mL
pHrodo BioParticles Conjugates	2 mg per vial	1 vial
**Total**	**1 mg/ml**	**2 ml**

Store at 4°C for up to 3 months.

**Table:** FluoroBrite DMEM medium

Reagent	Final concentration	Amount
FCS	10%	50 ml
FluoroBrite DMEM Medium	N/A	500 ml
**Total**		**550 ml**

Store at 4°C for up to 3 months.

***Alternatives:*** This protocol describes the usage of pHrodo BioParticles Deep Red Conjugates. However, you can use any other phagocytosis beads. You may need to include additional washing steps to remove beads and/or use different filter settings on the microscope. Adaptions are not necessary when using pHrodo BioParticles Deep Red Conjugates.

***Alternatives:*** We used FluoroBrite DMEM Medium, but you can use any buffered live cell imaging solution. Make sure that the medium does not contain fluorescent substances like phenol red.

***Alternatives:*** We used Dulbecco’s Phosphate Buffered Saline (PBS) and Fetal Bovine Serum South America (FCS), but any PBS and FCS which is used in your lab can be used.

***Alternatives:*** This protocol used primary murine microglia derived from C57BL/6 mice. However, you can use any phagocytosing cells of your interest.

***Alternatives:*** This protocol describes the usage of Cellvis 96-well plate with an optical glass bottom (#1.5), but you can use any plate system suitable for imaging. Remember that optical glass bottoms have the best optical quality. We did not test other plates; therefore, AI performance could be reduced when using plates with other optical parameters.

***Alternatives:*** We used the Olympus ScanR IX-83 high-throughput microscope. However, you can also use different microscopes if they are compatible to the Olympus ScanR software and if they are able to generate a 37°C 5% CO_2_ atmosphere for live cell imaging.

**CRITICAL:** You must use the Olympus ScanR software. Our provided neural network can only be imported into Olympus ScanR analysis software (v.3.4 or later) without any adaptions.

**CRITICAL:** Before using the pHrodo BioParticles Conjugates, use the sonicator bath to dissolve the particles for at least 15 min. The solution will get milky. Also refer to the latest Datasheet.

## Step-by-step method details

### Part 1: Set up the microscope and start pre-warming

**Timing: 1–2 h**

This first part of the protocol will adjust all the settings of the microscope including setting the correct objective and filters. Furthermore, we describe the necessary settings for pre-warming.

1. Turn on the microscope.

**CRITICAL:** This step is highly dependent on your system. Please follow the instructions on how to start the microscope of your core facility or local expert.

**Figure 1:**
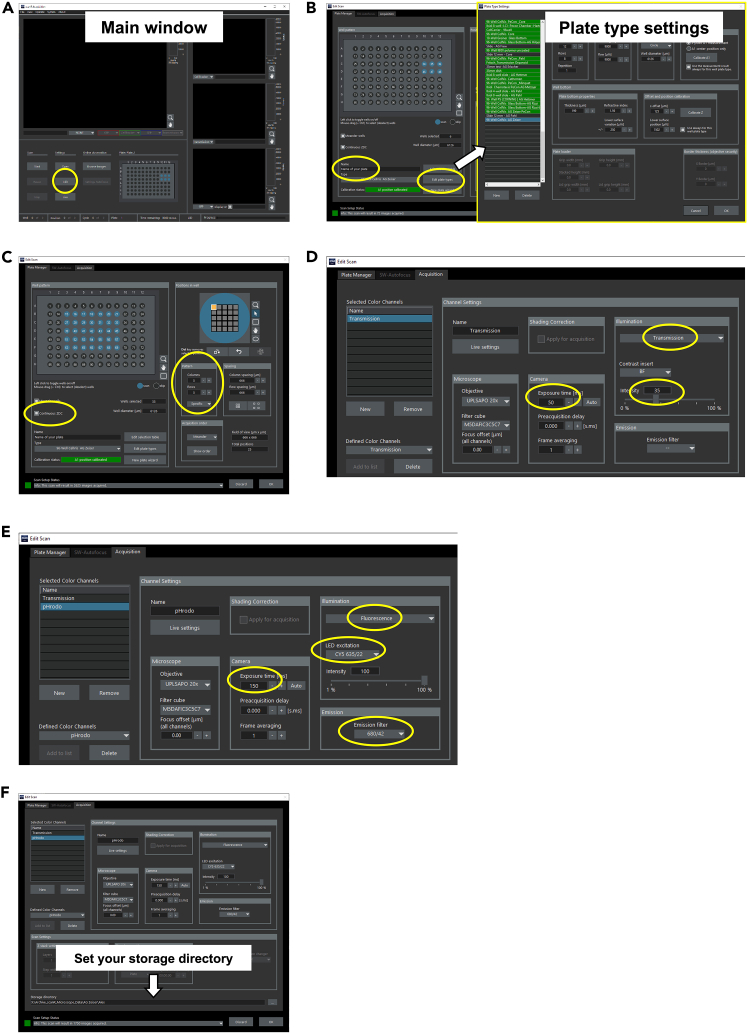
Setup of the microscope (A) Main window of the Olympus ScanR Acquisition Software. Edit-button encircled. (B) Edit scan window of the Olympus ScanR Acquisition Software. Plate name and Edit plate types-button encircled. Right panel shows the Plate type settings window. (C) Plate Manager tab of the Olympus ScanR Acquisition Software. Encircled: Continuous ZDC and imaging pattern. (D) Acquisition tab of the Olympus ScanR Acquisition Software with Transmission channel selected. Encircled: Exposure times and Illumination settings. (E) Acquisition tab of the Olympus ScanR Acquisition Software with pHrodo channel selected. Encircled: Exposure time, Illumination settings, excitation filter settings and emission filter settings. (F) Acquisition tab of the Olympus ScanR Acquisition Software. Storage directory highlighted.
  c. Set your Experiment name and press “Edit plate types” and select your plate and press “ok” (Figure 1B).
    **CRITICAL:** If you are the first person using a plate like the Cellvis 96-well glass bottom plate, ask your local expert how to set up and calibrate the plate you are using to avoid damage to the microscope and to your plate. A good calibration will enhance to focus and the quality of your images. To establish this protocol, we used an UPLSAPO 20x N.A. 0.75 objective. The neural network will work best at the same magnification.
  d. Select your wells, set continuous ZDC, and define how many images per well you want to image. We recommend using 5 × 5 images. (Figure 1C).
4. Set the correct excitation/emission filters.
  a. Go to the “Acquisition” Tab and set new channels:
    i. Transmission: This channel is necessary to use the neural network for cell detection. The settings can be seen in Figure 1D.
    ii. Press “New” and name the new channel “pHrodo”.
    iii. pHrodo: This channel is for the excitation and detection of the pHrodo bead signal. The settings can be seen in Figure 1E.
      **CRITICAL:** If you are using different conjugates of phagocytosis beads you may have to change the filter settings to achieve best excitation and detect the emission. Refer to the product data sheet for excitation and emission.
  b. Set your storage directory (Figure 1F).
  c. Press “ok”.
5. Start the pre-warming according to your local instructions.

***Note:*** This step is highly dependent on your system. Please follow the instructions of your core facility or local expert. Make sure to heat up to 37°C.

2. Open and boot the Olympus ScanR Acquisition software.
  a. Open the Olympus ScanR Acquisition software on your computer.
  b. Wait until the software boots.
  c. Press “ok” to calibrate the system settings.
3. Set the system settings.
  a. Place the proper plate holder for your plate on the microscope table.
  b. In the software, press “Edit” (Figure 1A).

**CRITICAL:** Pre-warming needs at least 1 h (depending on the insulation of your system). Proper pre-warming is essential for avoiding focus shifts during the live cell imaging due to temperature shifts.

### Part 2: Wash cells and add pHrodo BioParticles Conjugates

**Timing: 20 min**

In this part, we will prepare the cells for the live cell imaging of phagocytosis.

6. Put the pHrodo BioParticles Conjugates in the sonicator bath at 37°C for 15 min to disperse the particles in the PBS.
7. In the meantime, remove the culture medium from your cells and wash 2-3 times 1 min with FluoroBrite DMEM Medium (pre-warmed!). Add 200 μL per well for washing steps.
8. Add 100 μL FluoroBrite DMEM Medium per well.

**Pause point:** Incubate your cells in 100 μL FluoroBrite DMEM Medium, until the microscope pre-warming is done. You can also extend this period. In any case, make sure that you put the pHrodo BioParticles Conjugates in a bath sonicator for 15 mins prior to adding to the cells.

9. After pre-warming is done, take the pHrodo BioParticles Conjugates out from the bath sonicator and add 10 μL per well. Carefully resuspend once.

**CRITICAL:** Now, please proceed fast to the microscope as phagocytosis starts immediately.

**CRITICAL:** Keep your plate at 37°C to avoid water condensing which interferes with imaging. Ideally you have a sterile hood and an incubator near the microscope so that you can add the pHrodo BioParticles Conjugates there.

### Part 3: Mounting the plate and starting the imaging

**Timing: 3–12 h**

This part describes how to mount the plate on the microscope and how to do the fine adjustments of the focus and light path. It also describes how to start the live cell imaging cycle.

10. Open the climate chamber of your microscope.
11. Place your plate carefully in the correct orientation into the plate holder.
12. Close climate chamber immediately.

***Note:*** Make sure that you close the climate chamber properly to avoid temperature and CO_2_ drop.

**CRITICAL:** Make sure that you don’t see condensed water. If you see condensed water, see troubleshooting 1.

**Figure 2:**
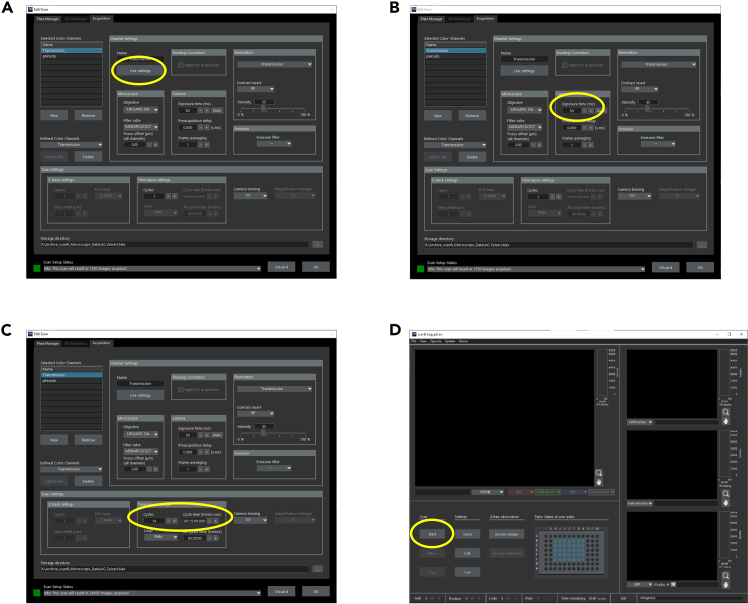
Adjustment of light paths and starting live cell imaging (A) Acquisition tab of the Olympus ScanR Acquisition Software. Live settings-button encircled. (B) Acquisition tab of the Olympus ScanR Acquisition Software. Encircled: exposure time. (C) Acquisition tab of the Olympus ScanR Acquisition Software. Encircled: Imaging cycles and Cycle time settings. (D) Main window of the Olympus ScanR Acquisition Software. Start-button encircled.
17. Check the focus.

***Note:*** If the focus needs improvements, see troubleshooting 2.

13. Go to the Olympus ScanR Acquisition software.
14. Press “Edit”.
15. Go to “Acquisition” tab.
16. Select Transmission and press “Live settings”. A new window should open (Figure 2A).

18. Adjust the exposure time of transmitted light to your satisfaction (Figure 2B).
19. Select pHrodo and press “Live settings”.
20. Reduce the exposure time so that there is only a low signal coming from your cells.

**CRITICAL:** A too high exposure time in the beginning will lead to oversaturation of the image over time as the signal intensity of the pHrodo BioParticles Conjugates will increase. Oversaturated images can cause problems in analysis, in any case, you are losing information.

21. After setting all exposure times, choose your preferred cycle time and numbers of cycles for the live cell imaging (Figure 2C).

***Note:*** Run a trial experiment to determine cycle time and number of cycles to capture the plateau phase of phagocytosis at the end of the live cell imaging. We used a cycle time of 15 min and 14 cycles.

**CRITICAL:** The minimal cycle time depends on the number of wells and images you want to acquire. The microscope needs to image all wells within the cycle time. Determine the minimal cycle time in your trial experiment.

22. Press “OK”.
23. Press “start”. The live cell imaging will start now (Figure 2D).

**Pause point:** You will now have time to clean up your working area and have a break. The microscope will run on its own for the selected time.

24. After the live cell imaging is done, remove your plate and shut down the microscope according to your local instructions.

***Note:*** Your data is automatically saved in your previously selected folder.

**Pause point:** Your data is saved in your selected folder. You can either directly proceed to Part 4 or you can take a break (variable).

### Part 4: Set up a new analysis assay using virtual channels

**Timing: 20 min**

Here we describe how to set up a new analysis assay using the virtual channel to detect the cells.

***Note:*** If you haven’t yet downloaded the Neural network from Mendeley Data (Zähringer, Alexander (2024), “Enhancing microglial phagocytosis analysis through deep learning: A U-Net based approach”, Mendeley Data, V1, https://doi.org/10.17632/czh96my8cf.1), please do so now.

***Note:*** You can use our sample assay provided on Mendeley Data (Zähringer, Alexander (2024), “Enhancing microglial phagocytosis analysis through deep learning: A U-Net based approach”, Mendeley Data, V1, https://doi.org/10.17632/czh96my8cf.1) to start with. In any case you may have to adjust the threshold (see step 34).

**Figure 3:**
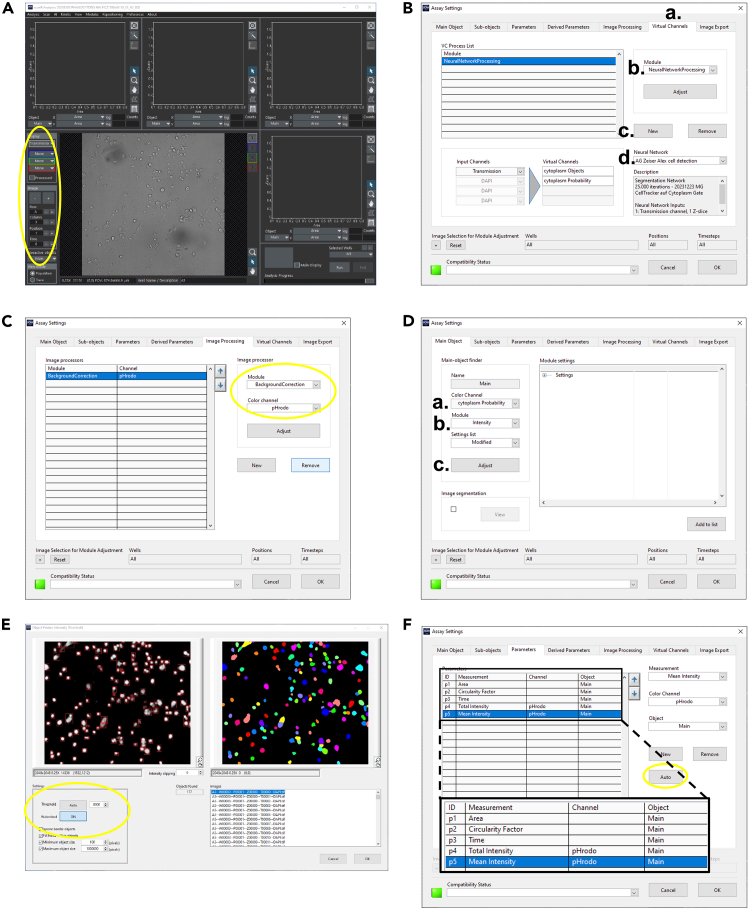
Setting up the analysis (A) Main window of the Olympus ScanR Analysis Software. Encircled: Control panel for images. (B) Virtual channels tab in the assay settings of the Olympus ScanR Analysis Software. (C) Image processing tab in the assay settings of the Olympus ScanR Analysis Software. (D) Main object tab in the assay settings of the Olympus ScanR Analysis Software. (E) Object Finder: Intensity Threshold in the assay settings of the Olympus ScanR Analysis Software. (F) Parameters tab in the assay settings of the Olympus ScanR Analysis Software. Zoom shows the necessary parameters.

***Note:*** If you do not see your images or some images are gray, see troubleshooting 3.

25. Transfer your imaging data to a powerful analysis storage.
26. Open Olympus ScanR analysis software.
27. Go to Scan – open and select your imaging data. You should now see an image of your scan and you can go through all images with the options on the left side next to the image (Figure 3A).

28. Go to Analysis – new Assay.
29. Go to AI – Manage Neural Networks – Import and import the downloaded neural network.
30. Go to Analysis – Assay settings, a new window opens.
31. Set the neural network as a new virtual channel:
  a. Go to “virtual channels” tab.
  b. Select “neural network processing” in “Module.
  c. Press “New”.
  d. Select the neural network in the drop-down menu (Figure 3B).
32. Go to “Image Processing” tab and select Background correction for pHrodo (Figure 3C).
33. Setting the Main object: The main object defines your cell.

***Note:*** If you also stained for the nuclei, we recommend using the nuclear staining as the main object and using the neural network as a sub-object to define the cytoplasm.

34. Go to the “Main object” tab.
  a. Select “cytoplasm probability” in “channels” (Figure 3D).
  b. Select “Intensity” (Figure 3D).
  c. Press “Adjust”, a new window opens (Figure 3D).
  d. Select “Watershed on”, this will split neighboring cells (Figure 3E).
  e. Adjust the threshold to your satisfaction. However, we recommend using the “Auto” function which will automatically set the threshold. The gray scale images in the top left corner will show you the object probability. The whiter, the higher the probability of being a cell (Figure 3E).
  f. From the list on the right side, select different images to see that your threshold matches all images. If you are using the “Auto” thresholding function, repeatedly execute this function on several images, since the threshold will change in between the images. We recommend using the lowest automatically determined threshold and reducing it by 1-5% and manually type in this value.

***Note:*** You can now also set sub-object definitions like cytoplasm or nucleus und the “Sub-objects” tab but this is not necessary for this protocol.

35. Go to “Parameters” tab and select “Auto”. This will result in a list of parameters of your main object. You can also manually select parameters if you want to further analyze your images (Figure 3F).

***Note:*** Make sure that the list of Parameters contains “Time” on Main object and “Total Intensity pHrodo” and “Mean Intensity pHrodo” on Main object. If these parameters do not appear in the list, see troubleshooting 4.

**Figure 4:**
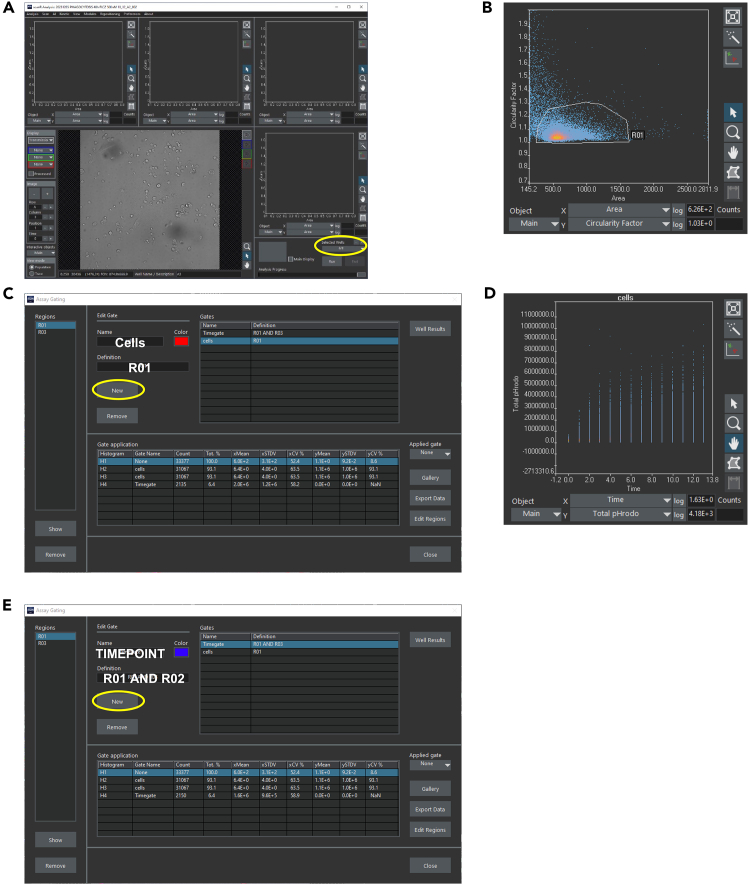
Gating of the detected cells (A) Main window of the Olympus ScanR Analysis Software. Encircled: Button for well-selection. (B) Top left plot showing the cell detection of one well. Each dot represents a detected object. (C) Assay Gating window of the Olympus ScanR Analysis Software for defining the “Cells”-gate. (D) Second plot showing time dependent increase in total intensity pHrodo (total pHrodo). Each dot represents a detected object. Remind the heading “cells” meaning that the “Cells” gate is applied to this plot. (E) Assay Gating window of the Olympus ScanR Analysis Software for defining the “Timepoint”-gate.

***Note:*** Depending on the computer hardware, this step can take some time.

***Note:*** You should now see dots in the plots, these are your detected cells. If this is not the case, see troubleshooting 5.

36. Close the window.
37. Go to Analysis – Save Assay, this will save the Assay we have now created.
38. To check whether the Assay works, select one well on the bottom left corner (Figure 4A) and press run.

39. To better understand what the neural network is doing, select “objects probability” as a color channel on the left side next to the image. The cells are now overlaid with an artificial staining.
40. Go to the first plot in the top left corner and select the following parameters:
  a. X = Area.
  b. Y = Circularity factor.
41. Now, draw a gate around your cell population as seen in Figure 4B.

**Figure 5:**
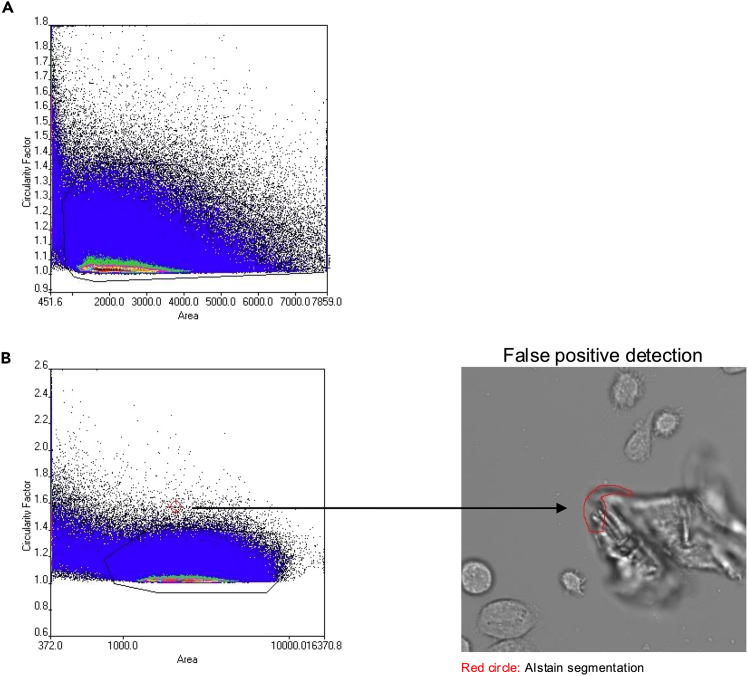
Alternative gating strategy and exclusion of false positive detections (A) Dot plot showing alternate cell gating strategy using Circularity factor and Area as cell definition parameters. For increased visibility, the background was changed to white. (B) Dot plot showing an example of cell gating. Note the different scaling of the X axis (log-scale) and its effect on the representation of the cell population. For increased visibility, the background was changed to white. Red crosshair pointing towards false positive detection as shown in the right image. Note the outlier localization in the dot plot.

***Note:*** The exact gating depends on the specific cells of interest. This protocol just gives an example how the gate will potentially look like. By clicking on a dot, your selected cell will be shown in the image below. Thereby you can adjust the gate.

***Note:***Figure 5A shows an alternate gating strategy and Figure 5B includes an example with how the gating could look like, including the indication of false positive detection. Gating is, unfortunately, subjective and user defined. One might have to figure out the best ways to identify their cells of interest and gate them accordingly. One might need some time to get a feeling for how the cells appear in the gates. However, we recommend using smaller gates that likely exclude some cells (e.g. draw a small gate around the “hot” area in the dot plot) thereby ensuring that no cell debris is used in the further analyses.

**CRITICAL:** The better your gate excludes cell debris or dead cells, the more accurate your results will be later.

42. Go to Analysis – Assay Gating and select “New” and insert the parameters as shown in Figure 4C to generate a gate called “Cells”.
43. Go to the second plot and right click set gate – Cells. Now only the cells inside your gate “Cells” will be shown in the second plot.
44. In the second plot, select the following parameters to show the time dependent phagocytic activity:
  a. X = Time.
  b. Y = Total intensity pHrodo.

***Note:*** You should now see a plot like in Figure 4D.

45. If you want the export data for each time point, go to the second plot and draw a new rectangular gate around any time point results (this gate will be called R02). You can drag and pull the gate to the time point you want to export the data later (Part 5).
46. Go to Analysis – Assay Gating and select “New” and insert the parameters as shown in Figure 4E to generate a gate called “Timepoint”.
47. Go to Analysis – Save Assay.

**Pause point:** You can take a break here and continue the analysis later.

### Part 5: Run the analysis

**Timing: 4+ h (variable)**

The last part describes the analysis process and how you will get your data.

48. Go to Analysis – Batch run, a new window will open.

***Note:*** Batch run enables you to run the analysis in the background and it will automatically save your data. Moreover, if you want to analyze more than one scan, you can queue them in the batch run.

**Figure 6:**
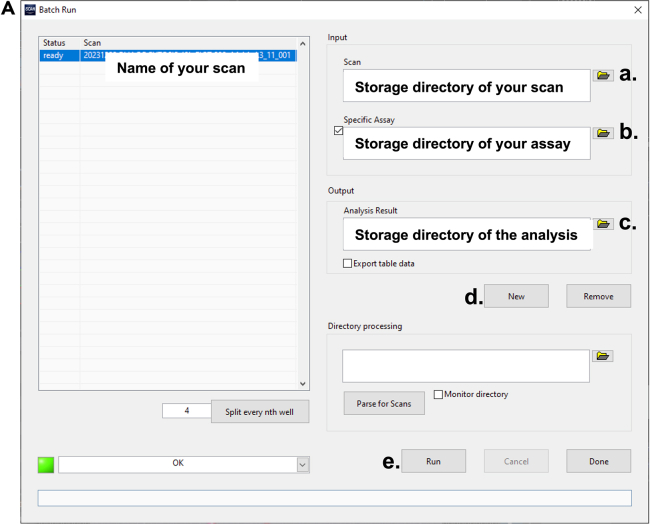
Starting a Batch run (A) Batch run window of the Olympus ScanR Analysis Software.

49. Start the Batch run (see also Figure 6):
  a. On the top right, select your scan.
  b. Then select your assay.
  c. Then select your storage directory. You may also rename the output file.
  d. Press “New”.
  e. Press “Run”.

**Pause point:** You can take a break now; the computer will analyze your scan.

***Note:*** The computing time depends on your computer hardware, network and amount of data.

For example: Analyzing 8 wells with 25 images per well and 14 time points will take approximately 4 h using the hardware specifications described above. The best time point to run the analysis will be overnight because your institutional network will likely be faster, and the computer is not blocked for and by other users of your lab.

50. Once the analysis is done, go to Analysis – open to open the analysis.
51. The second plot shows now the merge of all wells. If you only want to show one well at a time to take a snap of the graphics, select the appropriate well on the bottom left side of the main window.
52. If you want to export the numeric data at specific time points, set your time point gate in the second plot at the respective time point.
53. Go to Analysis – Assay gating – Well results and select “Timepoint” as the gate and the respective parameter you want to export.
54. Then click on export. A .txt file will be generated with the data per each well.
55. You can also export the data for each single cell by Analysis – Export.

***Note:*** Congratulations, you successfully ran and analyzed a live cell imaging phagocytosis assay!

### Adaptions for other cell types

If you want to use cell types other than primary microglia you might have to adapt this protocol at the following steps.

#### Part 2: Wash cells and add pHrodo BioParticles Conjugates

Depending on the ideal growth conditions of your cells, you should use other cell culture medium for incubating the cells on the microscope. Microglia grow well in DMEM medium, but e.g. some human macrophage cell lines may need RPMI medium for best growth conditions. You should check the datasheets of your cell lines for detailed information. Make sure that your medium does not contain any pH indicator like phenol red since these substances exhibit high autofluorescence.

#### Part 4: Set up a new analysis assay using virtual channels—Step 41: Now, draw a gate around your cell population...

The gating of the cell population is highly dependent on your cell type. The gating for microglia is shown in Figure 4B. However, if your cells are bigger, then your values for “Area” will be higher than shown in this protocol. Thereby you may need to drag the gate to higher values for “Area”. Additionally, “Circularity factor” changes with the complexity of the morphology. The more ramified your cells are, the higher the circularity factor will be and vice versa. You may need to play around with the gating, and you can visualize the gate by right click – show Gallery, which will lead to a new window showing you some cells inside the gate. You can use this function to adjust your gate.

There are no further adaptions of this protocol required. Independent of the cell type you are using, you need to plate 10.000 cells per well and add the same amount of beads as described above for microglia. The incubation time on the microscope (the duration of the live cell imaging) is individual for the experiment and the used experimental conditions e.g. drug treatments. The incubation needs to be determined in a pre-study.

#### Challenges when applying to other cell types

We tested AIstain on MV4-11 AML cells and JIMT-1 breast cancer cells which show a clearly different morphology than microglia. The neural network shows good performance on these cell types as well. However, AUC ROC values were slightly lower. The major challenge when applying this protocol to other cell types is that some cell lines like HEK-2 become confluent and do not respect other cell boundaries resulting in “merged” cells when running the neural network. Confluent cell lines should be avoided, or the cell density should be reduced to achieve a single-cell distribution on the plate.

### Downstream single-cell analysis

The Olympus ScanR software assigns an individual object ID to each detected object (= cell). For each single object, the software calculates the parameters which were selected by the researcher, e.g. area, circularity, pHrodo intensities. These parameters can be exported as a .txt-file and fed into further bioinformatic analyses. The software also allows assigning X and Y coordinates to each object and thereby allows plotting an intensity plot of each well or the whole plate. For using X and Y parameters select X and Y in the assay settings at step 35 (Figure 3F) from the drop-down menu.

The Olympus ScanR software itself does not provide this function. We attached an exemplary code for R below which can be used to generate an intensity plot using an Excel-file (import .txt-file into Excel):

library(ggplot2)

library(readxl)

library(ggpubr)

# Load the data

file_path <- "your_file.xlsx" # Replace with your actual file path

sheet_name <- "Sheet1" # Adjust as necessary

data <- read_excel(file_path, sheet = sheet_name)

# Ensure data contains the expected columns

if (!all(c("X", "Y", "Total Intensity pHrodo") %in% colnames(data))) {

stop("The Excel file must contain 'X', 'Y', and 'Total Intensity pHrodo' columns.")

}

# Create the intensity plot

plot <- ggplot(data, aes(x = X, y = Y, fill = `Total Intensity pHrodo`)) +

geom_tile() +

scale_fill_gradient(low = "blue", high = "red") + # Adjust colors as needed

theme_minimal() +

labs(title = "Phagocytosis Intensity Map",

 x = "X Coordinate",

 y = "Y Coordinate",

 fill = "Total Intensity pHrodo")

# Display the plot

print(plot)

Depending on the names of columns, you may need to update the column headings in the code in lines 11 and 16.

## Expected outcomes

A successful phagocytosis assay using this protocol should show a time dependent increase in total pHrodo intensity. The actual steepness and maximum of pHrodo intensity increase is variable as it differs between cell types and treatment conditions. In any case, you should see a pHrodo signal in your cells if you go through your acquired images. This means that your cells phagocytosed pHrodo BioParticles Conjugates. It is normal that you see increasing background signal over time as the pHrodo particles sediment. However, your fluorescent signal of the background should be low as the pHrodo BioParticles Conjugates only emit fluorescence if they reach the late endosome with a drop in the pH. Furthermore, the neural network should still recognize your cells.

**Figure 7:**
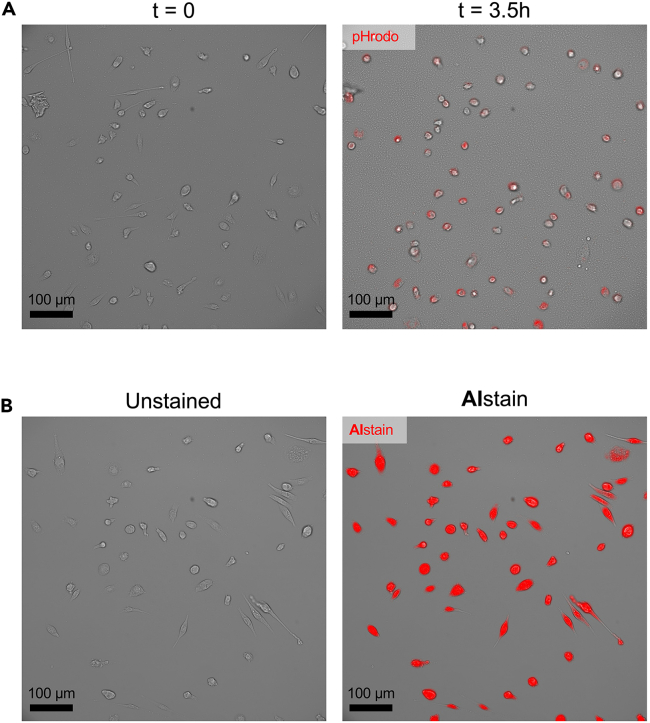
Expected outcomes (A) Representative images showing pHrodo (red) uptake of primary microglia at timepoint 0 h and after 3.5 h. Scale bar: 100 μm. (B) Representative image of unstained (left) and artificially stained (red, right) primary microglia. Scale bar: 100 μm.

An example of using this protocol for a phagocytosis assay of microglia is shown in Figure 7. As illustrated in Figure 4D, the Olympus ScanR analysis software shows a time dependent increase in Total intensity pHrodo in the second plot, reaching a plateau at time point 12. Representative images also show a time dependent increase in pHrodo uptake and a reduced phagocytic activity of the treatment group (Figure 7A).

The result of neural network cell detection and artificial staining is shown in Figure 7B.

As mentioned before, the neural network described in this protocol can also be used on fixed cells or other living cells and not only for phagocytosis analysis. We used this protocol from part 4 onwards for the definition of cytoplasm on fixed cells stained with DAPI and stained for phospho NF-κB for translocation studies. Therefore, you can use the virtual channel either for the main object or for sub-object definition. The analysis and the settings of the assay from part 4 onwards do not change when using fixed cells.

**Figure 8:**
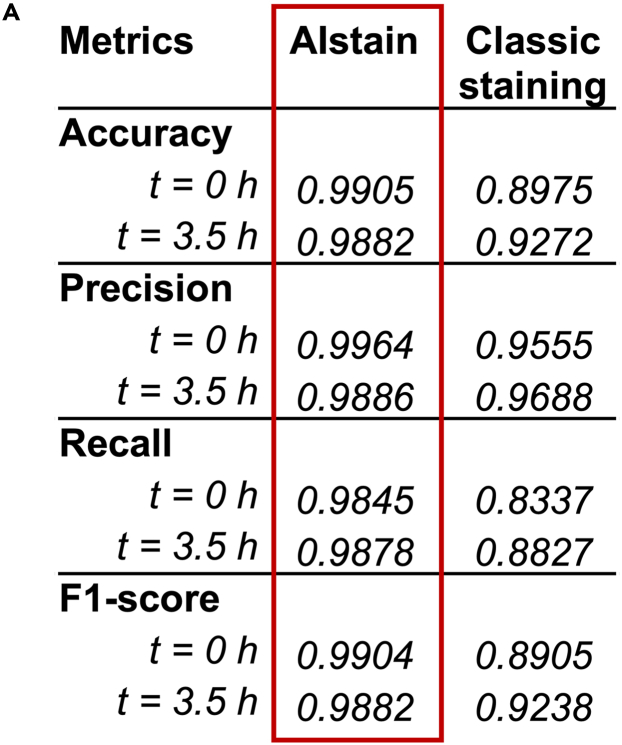
Performance metrics of AIstain and classic staining (A) Table showing the performance metrics of AIstain compared to classic fluorescent live cell staining using CellTracker.

An overview of the performance metrics of AIstain compared to classic fluorescent live cell staining using CellTracker are shown in Figure 8A. An example of use was published in Zähringer et al.^1^

## Limitations

While this deep learning-based protocol for live cell image cytometry analysis of microglial phagocytosis offers several advantages, it also presents specific limitations that must be considered.

The protocol is optimized primarily for microglia, which may limit its applicability to other cell types. Non-microglial cells may not be recognized with the same accuracy, potentially affecting the analysis. However, we have shown good detection rates in other cell types, specifically MV4-11 acute myeloid leukemia (AML) cells and JIMT-1 breast cancer cells as well. For using other cells types, follow the instructions above in adaptions for other cell types. Furthermore, the current protocol is designed for adherent cells; while the neural network can detect suspension cells, this capability requires further optimization of the protocol in the major parts 2 and 3.

The additional use of fluorescent dyes, necessary for certain analyses, introduces the risk of cytotoxicity and phototoxicity,^2^ which could alter cell behavior and thus affect experimental outcomes.^3^ Phototoxicity is a major challenge in live-cell fluorescence microscopy, primarily caused by the generation of reactive oxygen species (ROS) during light excitation. These ROS can damage cellular macromolecules such as DNA, proteins, and lipids, leading to morphological changes, impaired physiology, or even cell death.^2^ We therefore established the described phagocytosis assay without the need of fluorescent cell dyes, however, the pHrodo bioparticles are a source of fluorophores which can still cause phototoxicity. Furthermore, endogenous flavins can lead to phototoxicity. Moreover, a study by Stockley et al. showed that exposure with high doses of blue light changed microglia morphology with increased cell body volume,^4^ suggesting that microglia respond to light exposure independent of fluorescent labeling. In addition, Cheng et al. demonstrated that blue light affects gene expression of microglia.^5^ Our approach minimizes these effects by reducing the amount of blue light exposure by only acquiring bright-field images with low exposure times and Alexa Fluor 647 signal (excitation with 633 nm) which avoids the blue spectrum.

Another source of phototoxicity was found in riboflavin in DMEM culture media.^4^ There are formulations of culture media for neuronal and glial cultures without riboflavin and other phototoxic supplements provided by Stockley et al. We do not recommend using cell culture medium other than listed above in the key resources table for the microglia phagocytosis assay.

Moreover, extensive cell death within the sample can significantly compromise data quality. Dead cells may be inadvertently detected and analyzed by the neural network, leading to skewed results and inaccurate conclusions. Therefore, maintaining cell viability during experiments is crucial, and the potential impact of cell death must be carefully considered when interpreting the data obtained through this protocol.

A critical dependency of this protocol is the use of the Olympus ScanR system, which is integral to the successful application of this protocol. This reliance on specific hardware limits the generalizability of the approach, making it challenging to adapt the protocol to other imaging systems without significant modifications. Additionally, the computing time of the analysis is heavily hardware dependent.^6^ Variations in processing power, memory, and storage can significantly impact the time required to complete the analysis, potentially creating bottlenecks in laboratories without access to high-performance computational infrastructure. The deep learning models employed in this protocol are computationally intensive, requiring substantial resources that may not be readily available in all research settings.

Highly complex cells, with intricate morphology, are particularly challenging for the AI to capture accurately,^7^ leading to potential underrepresentation or misclassification in the analysis. There are several publications providing advanced segmentation tools for complex cells, underlining the difficulties of deep learning-based cell segmentation. MorphoSeg is an uncertainty-aware deep learning framework designed for the segmentation of complex cellular structures.^8^ It introduces a benchmark dataset featuring Ntera-2 (NT2) cells, a pluripotent carcinoma cell line, which exhibit diverse morphologies across multiple differentiation stages. By incorporating virtual outlier sampling during training, MorphoSeg enhances segmentation accuracy compared to state-of-the-art baselines.^8^ MARS-Net (Multiple-Microscopy-Type-Based Accurate and Robust Segmentation Network) is another deep learning pipeline developed to accurately segment cell edges and quantify cellular morphodynamics from live-cell imaging data.^9^ By utilizing transfer learning and data from multiple microscopy types, MARS-Net effectively localizes cell edges with high accuracy, making it suitable for analyzing complex cell morphologies.^9^ However, it should be noted that the Olympus ScanR environment does not yet support other models, such as MorphoSeg or MARS-Net. The present study proposes AIstain as a solution to this issue. AIstain is an easy-to-use U-Net-based neural network that has been implemented in the Olympus ScanR environment and shows high accuracy in detecting cells of different and complex morphologies.

Increased background noise further complicates cell segmentation,^10^^,^^11^ as it can obscure cell boundaries, leading to errors and potentially inaccurate data.

## Troubleshooting

### Problem 1

There is condensed water on the lid or at the bottom of the plate (related to Step 12).

Condensed water is the result of temperature differences between your culture medium and the plate as the plate got cold while transporting it to the microscope.

### Potential solution

Your plate maybe got cold. Try to incubate your plate for some time on the microscope in the climate chamber to equilibrate the temperatures. The condensed water should disappear. If this is not the case take a sterile wipe and wipe off the water.

### Problem 2

Image is not focused well (Related to Step 17).

Most often, this is caused by a wrong calibration of the plate and by temperature shifts.

### Potential solution

Make sure there is not Z-offset. Z-offset should be 0.

Make sure that the microscope is prewarmed properly.

If this did not improve the focus, go back to “Edit plate types” and select “Calibrate Z”. Set a new focus here.

### Problem 3

After opening the Scan in the Olympus ScanR Analysis software, some images cannot be viewed (related to Step 27).

This can happen if your data files cannot be read by the program or if some data got lost.

### Potential solution

• Make sure that you saved your imaging data in a directory where you have writing-rights. Sometimes directories are just for saving data but not for making changes in these data.
• Make sure you copied the complete imaging data folder with all the different files as metadata is necessary to read the images.

### Problem 4

Parameters are missing in the list in “Parameters” tab on Olympus ScanR Analysis software (related to Step 33).

Maybe the default settings on your system are different from ours, which leads to different default parameters.

### Potential solution

You can manually select the missing parameters by selecting them from the drop-down menu and pressing “new”. Make sure that you select all parameters as shown in Figure 3F.

### Problem 5

When starting the analysis in Olympus ScanR analysis software, you don’t see dots/cells appearing in the plots (related to Step 36).

This can happen if there are problems with the cell detection or calculation of parameters. Most of the times this problem is caused by wrong formulas in the derived parameters.

### Potential solution

Check all your settings in the assay again. Make sure that your formulas are correct. Try to run the analysis once again without any derived parameters. If the analysis is still not working, set up a new assay as described in part 4: set up a new analysis assay using virtual channels.

## Resource availability

### Lead contact

Further information and requests should be directed to and will be fulfilled by the lead contact, Robert Zeiser (robert.zeiser@uniklinik-freiburg.de).

### Technical contact

Technical questions on executing this protocol should be directed and will be answered by the technical contact, Alexander Zähringer (alexander.zaehringer@uniklinik-freiburg.de).

### Materials availability

This study did not generate new unique reagents.

### Data and code availability

The training data set for the neural network is available on Mendeley Data (Zähringer, Alexander (2024), “Enhancing microglial phagocytosis analysis through deep learning: A U-Net based approach”, Mendeley Data, V1, https://doi.org/10.17632/czh96my8cf.1).

The neural network is available on Mendeley Data (Zähringer, Alexander (2024), “Enhancing microglial phagocytosis analysis through deep learning: A U-Net based approach”, Mendeley Data, V1, https://doi.org/10.17632/czh96my8cf.1).

## Acknowledgments

This study was supported by the Deutsche Forschungsgemeinschaft (DFG, German Research Foundation)—SFB-1479—Project ID 441891347 (P01, S02 to R.Z.), Project ID 259373024—TRR 167 (to R.Z.), the European Union: EU Proposal n°ERC-2022-ADG Project 101094168—AlloCure (ERC Advanced Grant to R.Z.), ERA-NET Transcan – PIXEL (to R.Z.), ERA-NET Transcan – SmartCAR-T (to R.Z.), the Germany’s Excellence Strategy (CIBSS – EXC-2189 – Project ID 390939984 to R.Z.), the MOTI-VATE program of the Medical Faculty, Albert-Ludwigs-University of Freiburg (A.Z.), the Deutsche Krebshilfe (grant number 70114655), the Jose-Carreras Leukemia Foundation grant number DJCLS 09R/2022 (R.Z.), EU Project 101119855—exTra, and Leukemia & Lymphoma society (LLS Grant ID: 7030-23 to R.Z.). The project was supported by the Lighthouse Core Facility (Medical Faculty, University of Freiburg 2023/A2-Fol and 2021/B3-Fol; DFG Project ID 450392965). This study was funded by the Hans A. Krebs Medical Scientist Program, Faculty of Medicine, University of Freiburg (to J.M.V.).

## Author contributions

Conceptualization and investigation were done by A.Z. and M.F. Writing of the original draft was done by A.Z. Writing, review, and editing were done by J.M.V., M.F., T.W., and R.Z. R.Z. and M.F. supervised the project.

## Declaration of interests

R.Z. has received honoraria from Novartis, Incyte, Sanofi, Medac, Neovii, and Mallinckrodt.
